# Histone deacetylase inhibitors as a novel therapeutic approach for pheochromocytomas and paragangliomas

**DOI:** 10.32604/or.2022.026913

**Published:** 2023-02-03

**Authors:** ASPASIA MANTA, SPYRIDON KAZANAS, STEFANOS KARAMAROUDIS, HELEN GOGAS, DIMITRIOS C. ZIOGAS

**Affiliations:** 1 Endocrine Unit, Second Department of Internal Medicine Propaedeutic & Research Institute, Medical School, National and Kapodistrian University of Athens, Attikon University Hospital, Athens, 12461, Greece; 2 First Department of Internal Medicine, Medical School, National and Kapodistrian University of Athens, Laikon General Hospital, Athens, 11527, Greece; 3 Department of Obstetrics & Gynecology, General Hospital of Elefsina Thriassio, Elefsina, 19600, Greece

**Keywords:** HDACis, HDAC inhibitors, Neuroendocrine tumors, Epigenetics, Histone deacetylation, Cancer

## Abstract

Epigenetic mechanisms, such as DNA methylation and histone modifications (e.g., acetylation and deacetylation), are strongly implicated in the carcinogenesis of various malignancies. During transcription, the expression and functionality of coding gene products are altered following the histone acetylation and deacetylation. These processes are regulated by histone acetyltransferases (HATs) and histone deacetylases (HDACs), respectively. HDAC inhibitors (HDACis) have been developed as promising therapeutic agents, to limit exposure to traditional and toxic chemotherapies and offer more alternatives for some specific malignant diseases with limited options. Mechanistically, these agents affect many intracellular pathways, including cell cycle arrest, apoptosis and differentiation, and their mechanism of action mainly depends on the type of cancer. Currently, five HDACis have been approved for the treatment of several hematological malignancies (e.g., T-cell lymphoma subtypes and multiple myeloma); while, many of them are tested for further therapeutic indications in solid tumors (e.g., colorectal, thyroid, breast, lung and pancreatic cancer). Herein, we review the literature and gather all available evidence, from *in vitro* and *in vivo* data to clinical trial results, that recognizes the antitumor activity of HDACis on pheochromocytomas and paragangliomas; and supports their clinical implementation in the treatment of these rare neuroendocrine tumors at metastatic setting.

## Introduction

Pheochromocytomas (PCCs) and paragangliomas (PGLs) are rare neuroendocrine tumors that arise from chromaffin cells and frequently secrete one or more catecholamines. Pheochromocytomas arise from the adrenal medulla, whereas PGLs originate from extra-adrenal sympathetic or parasympathetic ganglia [1]. The diagnosis may be disregarded during life and be discovered in 0.05%–0.1% of autopsies [1]. Usually, PCCs are detected by chance (21.1%–57.6%) and constitute approximately 4%–8% of all adrenal incidentalomas [2,3]. Updating the previous epidemiological data where 10% of PCCs/PGLs were identified as malignant [4], all PCCs/PGLs are now considered potentially metastatic [5], and all patients should be advised for genetic counseling [1]. Currently, 25%–30% or more of these tumors are attributed to genetic background [6]; at least 15 PCC/PGL-related genes have been recognized, and 12 syndromes have been described [7].

The diagnosis of PCC/PGL is based on detecting urinary metanephrines [8]. Following the biochemical diagnosis, CT scanning should be performed [1]. At the same time, functional imaging should also be used in suspicion of metastatic disease, including positron emission tomography (PET)/CT with various radiotracers [9] and 123I-metaiodobenzylguanidine (123I-MIBG) scintigraphy, especially to recognize those patients that could also be treated with 131I-MIBG [10]. After a multidisciplinary team consideration, most PCCs and PGLs can be treated surgically [1]. Still, for some unfit cases, therapy with 131I-MIBG could also be a reasonable option, if 123I-MIBG scintigraphy is positive [11,12]. For metastatic PCCs/PGLs, different combinations of conventional chemotherapy, mainly the regimen cyclophosphamide, vincristine and dacarbazine, have been used for many years [13–15], but recently, novel agents, including tyrosine kinase inhibitors [16], somatostatin analogs [17], hypoxia-inducible factor (HIF) inhibitors, mTOR inhibitors, histone deacetylase inhibitors (HDACis), DNA-alkylating agents and immune checkpoint inhibitors [18] are under testing for the treatment of metastatic/unresectable setting of these rare neuroendocrine tumors.

Among those approaches, the inhibition of histone deacetylation via HDACis has been entered into the focus of this study. The imbalance between histone acetylation and deacetylation can epigenetically change the expression of tumor suppressor genes and/or proto-oncogenes [19–21], that control cancer evolution and progression [22,23]. So far, 18 human HDACs have been identified into two families according to the implicated co-factor [24–26]. Different classes of HDACs are located in different cellular compartments [23,27]. An overview of the classification of human HDACs, their cellular localization and their tissue expression is presented in Table 1 [25–28].

**Table 1:** HDAC classification, cellular localization and tissue expression

HDAC classes				Cellular localization	Normal tissue expression
**Classical HDAC family** *Zn* ^*2+*^ *-dependent*	**Class I**		HDAC1	Nucleus	All tissues
			HDAC2	Nucleus	All tissues
			HDAC3	Nucleus	All tissues
			HDAC8	Nucleus, Cytoplasm	Smooth muscle
	**Class II **	**IIA**	HDAC4	Nucleus, Cytoplasm	Brain, heart, skeletal muscle
			HDAC5	Nucleus, Cytoplasm	Brain, heart, skeletal muscle
			HDAC7	Nucleus, Cytoplasm	Heart, lungs, placenta, pancreas, skeletal muscle, thymus
			HDAC9	Nucleus, Cytoplasm	Brain, skeletal muscle
		**IIB**	HDAC6	Cytoplasm	Heart, brain, skeletal muscle
			HDAC10	Nucleus, Cytoplasm	
	**Class IV**		HDAC11	Nucleus	
**Sirtuin family ** *NAD* ^*+*^ *-dependent*	**Class III**		SIRT1	Nucleus	
			SIRT2	Cytoplasm	All tissues
			SIRT3	Mitochondria	All tissues
			SIRT4	Mitochondria	All tissues
			SIRT5	Mitochondria	All tissues
			SIRT6	Nucleus	All tissues
			SIRT7	Nucleus	All tissues

**FIGURE 1:**
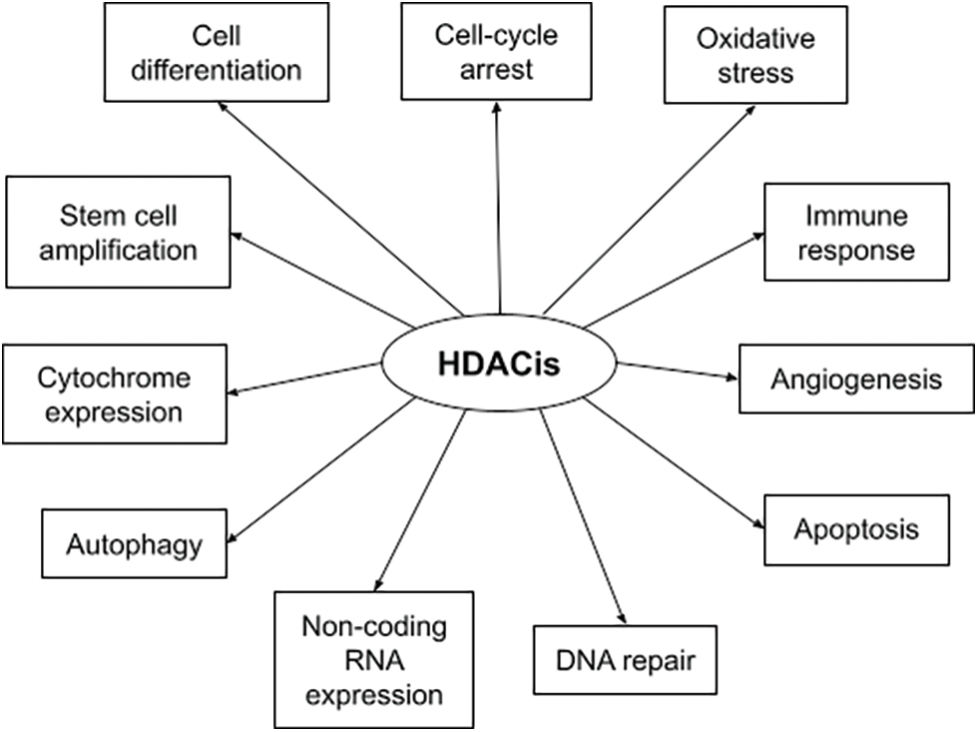
Summary of HDACis’ mechanisms of action in cancer.

HDACs are overexpressed in hematologic and solid malignancies, and their inhibition became a promising anti-cancer theory. HDACis include both natural and synthetic compounds. Some HDACis selectively inhibit specific HDAC classes while others are pan-HDAC inhibitors [29]. HDACis increase acetylation of both histone and non-histone proteins to a significant degree, resulting in cell cycle arrest, cell differentiation, induction of cell death/apoptosis (e.g., oxidative stress generation, disruption of mitosis and mitotic cell death, autophagy, etc.), as well as blocking of angiogenesis [30]. Fig. 1 depicts the multiple mechanisms of action of HDACis [29]. The main HDACis under clinical testing, their targeted HDACs, their chemical nature and their approved indications are presented in Table 2 [23,29,31,32]. The antitumor activity of these agents depends on the specific type and stage of cancer, the characteristics of each patient and the administered dose [23,33].

**Table 2:** HDACis under clinically testing as anticancer agents

HDACi	HDAC Target	Chemical Class	Cancer Type	FDA Approval
Vorinostat	Class I, II and IV	Hydroxamic acid	CTCL, Melanoma, Gastric, Breast, NSCLC, Ovarian, Thyroid	CTLC
Belinostat	Class I, II and IV	Hydroxamic acid	PTCL, Breast, Ovarian, SCLC, Neuroendocrine	PTLC
Panobinostat	Class I, II and IV	Hydroxamic acid	MM, Breast, CML, SCLC, Prostate, Nasopharyngeal, Renal, Melanoma	MM
Pracinostat	Class I, II and IV	Hydroxamic acid	AML, Prostate	
Givinostat	Class I and II	Hydroxamic acid	CLL, HL, MM	
Resminostat	Class I and II	Hydroxamic acid	Colorectal, HCC, HL, CTCL	
Abexinostat	Class I and II	Hydroxamic acid	CLL, HL, NHL, Breast, Melanoma, Sarcoma, Renal, DLBCL	
Quisinostat	Class I and II	Hydroxamic acid	CTCL, Leukemia, NSCLC, Ovarian, MM,	
Nanatinostat	Class I	Hydroxamic acid	Nasopharyngeal, EBV-Related Tumors	
Rocilinostat	HDAC 6	Hydroxamic acid	MM, Breast, CLL, Cholangiocarcinoma	
Mocetinostat	Class I and IV	Benzamide	Solid Tumors, Melanoma, NSCLC	
Domatinostat	Class I	Benzamide	Advanced Hematologic Malignancies	
AR-42	Class I, II, and IV	Hydroxamic acid	MM, AML	
Entinostat	Class I	Benzamide	Solid Tumors, Melanoma, Lymphoma, AML, Breast, Colon	
Alteminostat	Class I and II	Hydroxamic acid	DLBCL, MM	
Tacedinaline	Class I	Benzamide	Lung, Pancreatic, MM	
Tucidinostat/ Chidamide	HDAC 1, 2, 3, 10	Benzamide	Breast, PTCL, Cervical, Gastric, Esophageal, NSCLC	PTCL
Romidepsin	Class I	Cyclic peptides	CTCL, PTCL, HL, NHL, Breast	CTLC, PTLC
Valproic Acid	Class I and II	Fatty acid	Hematologic & Solid Tumors, CLL, Brain	
Sodium butyrate	Class I and II	Fatty acid	Hematologic & Solid Tumors	
Pivanex	Class I and II	Fatty acid	NSCLC, MM, CLL	
Nicotinamide	Class III	Sirtuins Inhibitor	Laryngeal, Bladder, NSCLC	

Abbreviations: CTCL: Cutaneous T-cell Lymphoma; PTCL: Peripheral T-cell Lymphoma; DLBCL: Diffuse Large B Cell Lymphoma; MM: Multiple Myeloma; AML: Acute Myeloid Leukemia; HCC: Hepatocellular Carcinoma; HL: Hodgkin Lymphoma; NHL: Non-Hodgkin Lymphoma; CML: Chronic Myeloid Leukemia; CLL: Chronic Lymphocytic Leukemia; NSCLC: Non-Small-Cell Lung Carcinoma.

In this review, we summarize the research background and the development status of currently tested HDACis for treating metastatic/unresectable PCCs/PGLs and gather all published evidence from *in vitro* and *in vivo* studies up to clinical trials, supporting their implementation in oncological practice. The most important clinical trials investigating the use of HDACis in the treatment of PCCs/PGLs and NETs are presented in Table 3.

**Table 3:** Major clinical trials investigating the use of HDACis in the treatment of advanced PCCs/PGLs and NETs

Study	Type	Agent(s) used	Design	No. of patients	Diagnosis	Result	Comments
Mohammed et al., 2011 [34]	Phase II	**Valproic acid**	Twice daily PO	8	Low-grade NET	Stabilization of progressive disease and improvement of NET biomarker chromogranin A. Increase of Notch-1 post-treatment.	Patients with PCC/PGL excluded.
Fu et al., 2015 [35]	Phase I	**Vorinostat** + pazopanib	600 mg daily of pazopanib + 300 mg daily of vorinostat	78 (1 PCC)	Advanced solid tumors	No meaningful antitumor activity overall.	Patients with a hotspot T53 mutation had a notably better response.
Kelly et al., 2005 [36]	Phase I	**Vorinostat**	400 mg once daily/200 mg twice daily/300 mg twice daily for 3 days/week	73 (1 PGL)	Advanced solid and hematologic malignancies	Proposed dosing schedules safe for prolonged treatment, sufficient inhibition of HDAC activity.	Only 30% of patients remained in the study for 4 or more months.
DuBois et al., 2015 [37]	Phase I multicenter	**Vorinostat +** ^131^I-MIBG	Oral vorinostat once daily on days 1–14, intravenous ^131^I-MIBG on Day 3	27	Relapsed or refractory neuroblastoma	Histone acetylation increased post-treatment. MTD determined at 180 mg/m^2^/dose for vorinostat and 18 mCi/kg for MIBG.	Potentially useful results for other tumors treated with ^131^I-MIBG.
Pollard et al, 2021 [38]	Pilot study	**Vorinostat**	4 days of vorinostat followed by imaging with ^123^I-MIBG and ^68^Ga-DOTATOC	50	Metastatic midgut NET	Significant increase in ^68^Ga-DOTATOC uptake in some liver metastases.	Further research for pretreatment with HDACis needed.
DuBois et al., 2021 [39]	Phase II	^131^I-MIBG, ^131^I-MIBG + Vincristine + Irinotecan, ^131^I-MIBG + **Vorinostat**	One course of treatment-Group A: ^131^I-MIBG only, Group B: ^131^I-MIBG + IV Vincristine + IV Irinotecan, group C: ^131^I-MIBG + Vorinostat orally once daily	105	Relapsed or refractory neuroblastoma	The group treated with ^131^I-MIBG + Vorinostat had the best response rate with minimum toxicity.	Potentially useful results for other tumors treated with ^131^I-MIBG.
Balasubramaniam et al., 2018 [40]	Phase I	**Belinostat** + cisplatin + etoposide	48 h continuous IV infusion of belinostat	28 (2 PCC/ PGL)	Advanced NETs and SCLC	Safe dose for future studies confirmed at 500 mg/m^2^/24 h. In patients with PCC/PGL, the treatment resulted in one stable disease after 4 cycles of treatment and one progressive disease after 2 cycles of treatment.	A specific group of patients with the UGT1A1 genotype was notably affected by AEs.
Jin et al., 2016 [41]	Phase II	**Panobinostat**	Oral panobinostat 20 mg once daily 3 days/week	15	Metastatic low-grade NETs	High stable disease rate, median progression free survival of 9.9 months. Treatment relatively well tolerated.	Different NETs possibly respond differently to therapeutic agents.

## HDACis in Pheochromocytomas & Paragangliomas

### Valproic acid (VPA)

Valproic acid is a branched short-chained fatty acid that was synthesized in 1882 and was approved for treating epilepsy in 1967 [42]. Since then, it has been used as an effective anticonvulsant medication in many neurological indications. After discovering that VPA can inhibit both HDAC classes I and IIA, many *in vitro* studies and clinical trials examined its use in different tumor types, including ovarian, breast, lung, pancreatic and thyroid cancer [43,44]. Adler et al. observed that rat PCC cells treated with increasing doses of VPA reduced their neuroendocrine tumor (NET)-related biomarkers, achaete-scute complex-like1 (ASCL1) and chromogranin A (CgA), and subsequently decreased their hormone secretion. The authors concluded that VPA-treated cells suppressed their growth rate by nearly 70% compared to controls in a dose-dependent manner by activating apoptotic pathways, mainly Notch-1 signaling [45]. Notch-1 is inactive in NETs, and its activation is associated with tumor growth inhibition and analogical decrease in NET-related biomarkers [46]. In another experiment in rat PCC cells, treatment with VPA for 48 h showed a similar dose-dependent effect on HDAC-mediated cancer cell growth [47]. In the clinical setting, an interesting phase II study including patients with low-grade neuroendocrine neoplasms was published in 2011 (Table 3). Although patients with PCC/PGL were excluded from this study, treatment with VPA resulted in stable disease in most of included patients, improving the levels of chromogranin A. It is worth noting that post-treatment Notch-1 was upregulated by ten times on average, in the VPA-treated population [34].

### Vorinostat (Suberanilohydroxamic acid–SAHA)

Vorinostat is an antitumor agent with inhibitory effects on both classes of HDACs (I and II). At doses that have little or no toxicity on normal cells, it was found that it can cause growth arrest and cell death [48] and subsequently, its therapeutic efficacy was examined as a monotherapy or in combined regimens with promising results [48,49]. Since October 2006, vorinostat has received approval from FDA for patients with cutaneous T-cell lymphoma (CTCL) who have progressive, persistent or recurrent disease on or following two prior systemic therapies [50]. Repositioning some new therapeutic options to the metastatic PCC/PGL, Giubellino et al. suggested that the synergistic combination of the HDAC inhibitor SAHA and the topoisomerase inhibitor epirubicin could represent an example of possible successful treatment [51]. In patients with PCC/PGL, a mutation in the mitochondrial complex II subunit (succinate dehydrogenase subunit B, SDHB) is associated with more aggressive and extensive disease [52,53]. Yang et al. showed that SDHB expression was reduced in SDHB-mutated tumors, and the administration of vorinostat could prevent further degradation and restore the quantity of functional SDHB, blocking the proliferative signaling [54]. The effect of vorinostat on advanced solid and hematologic malignancies has been examined by 3 phase I and 1 phase II clinical trials as well as by a pilot study on imaging of metastatic midgut NET (Table 3). The dosing schedules were safe with sufficient inhibition of HDAC activity either as vorinostat monotherapy or in a combination with other agents, including pazopanib and [131I]-MIBG. Overall, the efficacy results from these trials were modest [35,38], and only Kelly et al. [36] achieved keeping 30% of patients with advanced solid and hematologic malignancies in the study for more than four months [47–50]. Recently, a randomized phase II clinical trial compared 131I-MIBG plus vorinostat *vs*. 131I-MIBG alone and *vs*. 131I-MIBG plus vincristine plus irinotecan in 105 patients with neuroblastoma. The combination of 131I-MIBG plus vorinostat offered the highest response rate [39].

### Belinostat (PXD101)

Belinostat is a hydroxamic acid that was approved for treating peripheral T-cell lymphoma (PTCL) in 2014. It is currently under investigation for the treatment of both hematologic and solid malignancies, as monotherapy or in a combination with other anticancer agents [23,29]. It is considered a pan-HDACi, blocking Class I, II and IV HDACs [22]. Recently, a prodrug of belinostat, ZL277, was reported to be superior in bioavailability and efficacy. Because of its superior biocompatibility and intratumoral penetration, ZL277 was found to be more effective than belinostat *in vivo*, not only preventing tumor development but also dramatically lowering tumor sizes in an MCF-7 xenograft tumor model [55]. However, these results are preliminary enough, and more studies are needed to support its potential use in humans [52]. A phase I clinical trial tried to determine the maximum tolerated dose of the combination of belinostat (in a 48-h continuous IV infusion on days 1–2, reached 500 mg/m^2^/24 h) with cisplatin (a 1-h IV infusion of 60 mg/m^2^ on day 2), and etoposide (a 1-h IV infusion of 80 mg/m^2^ on days 2, 3, and 4) in 28 patients with advanced small cell lung cancer (SCLC) (Table 3). The combination was safe and active in SCLC and other neuroendocrine cancers. Objective responses were observed in 11 (39%) of 28 patients and seven (47%) of 15 patients with neuroendocrine tumors. In the 2 included patients with PCC/PGL, the combination resulted in one stable disease after four cycles, and one progressive disease after two cycles of treatment Patients carrying more than three copies of variant UGT1A1 (*28 and *60) had higher serum levels of belinostat because of slower clearance. Future phase II studies incorporating the genotyping information for UGT1A1*28 and UGT1A1*60 are needed to identify candidates for this combination [40].

### Sodium butyrate (NaB)

Sodium Butyrate is a short-chain fatty acid and one of the oldest identified HDACis [56,57]. It is typically produced in the gastrointestinal tract through anaerobic bacterial fermentation of dietary fibers. Among the fatty acids, NaB displays the most remarkable efficacy in inhibiting HDAC activity, including most HDAC classes. Many studies have examined its use in different types of cancer. Primarily due to its production in the colon, NaB may have a protective role in the development of colon cancer [58,59]. The impact of NaB in rat PCC cells was initially described in 1987, when Byrd et al. observed that treatment with NaB ceased cell division and altered the cellular “malignant” phenotype [56]. In rat PCC cells, NaB reduced cell growth and the levels of NET biomarkers ASCL1 and CgA in a dose-dependent manner, activating the Notch-1 pathway and subsequent carcinogenesis, as mentioned above. The tumor growth was inhibited due to the concurrent arrest of the cell cycle and the induction of apoptosis [60].

### Trichostatin A (TSA)

Trichostatin A is a metabolic compound isolated from the strains of *Streptomyces hygroscopicus* in 1975 primarily used for its antifungal activity [61]. TSA displays a similar inhibitory activity in Classes I and II HDAC, keeping some slight differences among particular HDAC counterparts, but remains ineffective in Class III HDAC [62,63]. It has shown anti-proliferative properties, inducing cell cycle arrest, differentiation and apoptosis [64]. Treatment with TSA inhibited the proliferation of mouse pheochromocytoma cells (MPC) in a dose- and time-dependent manner, while increased specific [3H]-norepinephrine and 123I-MIBG uptake. *In vivo* experiments showed that TSA-treated tumor-bearing mice presented an increased uptake of 123I-MIBG and 18F-fluorodopamine in their metastatic liver lesions. Although TSA may enhance the response to 131I-MIBG treatment and make more effective the123I-MIBG-mediated diagnosis of metastatic disease, its poor *in vivo* availability will never permit its use in clinical trials in patients [65].

### Romidepsin (FR901228 or FK228)

Romidepsin is a bicyclic depsipeptide that was isolated from *Chromobacterium violaceum* cultures, first reported in the literature in 1994 [66]. In 1998, Nakajima et al. showed that romidepsin could inhibit intracellular HDAC. Its mechanism of action is similar to TSA, despite their chemical and structural differences [67]. Romidepsin was found to inhibit the growth of tumor cell lines, but its anticancer efficacy varied against different tumor tissues [68]. Currently, romidepsin has been approved for treating patients with CTCL or PTCL who have received at least one prior line of therapy [25]. Martiniova et al. found that *in vitro* exposure of MPC cells to romidepsin increases the uptake of 3H-norepinephrine, 18F-fluorodopamine and 123I-MIBG and causes a dose- and time-dependent decrease of cell proliferation. Further *in vivo* studies revealed that mice with metastatic PCC treated with romidepsin had increased uptake of 123I-MIBG and 18F-fluorodopamine in their liver lesions. Taken together these results, we could suggest that romidepsin may work as a useful diagnostic and therapeutic tool, improving the accuracy of 123I-MIBG scintigraphy or 18F-fluorodopamine PET, and in parallel, increasing the response of 131I-MIBG treatment in patients with PCC [65].

### Suberoyl bis-hydroxamic acid (SBHA)

Suberoyl bis-hydroxamic acid is a close analog of SAHA that acts as a HDACi and has been tested in treating NETs. It activates Notch-1 signaling, suppresses the secretion of NET-related biomarkers and hormones and inhibits cell proliferation by inducing cell cycle arrest or apoptosis in various cancer cell lines, including gastrointestinal and pulmonary carcinoid cells and medullary thyroid cancer cells [69–71]. Adler et al. treated rat PCC cells with gradually increasing doses of HDACis. Treatment of PCC cells with SBHA had similar results as treatment with VPA. The NET biomarkers ASCL1 and CgA were decreased. SBHA in a dose of 40μM suppressed tumor growth in more than 70% of PCC cells after six days of treatment and activated the apoptotic pathway. More specifically, the Notch-1 signaling pathway was upregulated 3-fold upon treatment with 40 μM of SBHA [45].

### *(−)-*Epigallocatechin-3-gallate (EGCG)

(−)-Epigallocatechin-3-gallate (EGCG) is one of the most substantial polyphenolic extracts in green tea. It has been reported that EGCG inhibits DNA methyltransferase and reactivates methylation-silenced genes in various cancer cell lines (e.g., human colon cancer HT-29 cells, esophageal cancer KYSE 150 cells, and prostate cancer PC3 cells) [72]. This induced inhibition of DNA methylation by a commonly consumed dietary constituent suggested a potential use of EGCG for the reversal of related gene silencing in the prevention of carcinogenesis. EGCG acts in a concentration-dependent manner affecting class I HDACs, a especially HDAC1 [73,74]. Hu et al. studied the effect of EGCG on PCC-xenografted mice. Treatment with EGCG affected both tumor growth and apoptosis via activating the caspases 3 & 7 and decreasing amyloid precursor protein (APP) levels [75]. This specific APP protein seems to play a significant role in various diseases, including Alzheimer’s disease. It has been studied as a diagnostic tumor biomarker or as a targetable molecule in distinct cancer types, including pancreatic adenocarcinoma or colon carcinoma. Except EGCG, studies have shown that also other HDACis (such as VPA) could downregulate the levels of APP, leading to decreased tumor growth, invasion and angiogenesis [76].

### Panobinostat

Panobinostat is a pan-HDAC inhibitor with high efficacy that has shown substantial antineoplastic activity in various cancer cell lines and is currently being clinically tested against hematologic and solid malignancies [77]. In 2015, it was approved for treating multiple myeloma in patients who have received at least two previous treatments [78]. A Phase II clinical trial showed that treatment with panobinostat resulted in a high rate of stable disease and a median progression-free survival (PFS) of 9.9 months with tolerable toxicity in patients with metastatic low-grade neuroendocrine tumors. Still, no data is available regarding PCCs/PGLs (Table 3) [41].

## Conclusion

PCCs and PGLs are rare neuroendocrine tumors with complicated genetic backgrounds and an unmet need for a more individualized approach, especially in the metastatic or unresectable setting. The benefits from conventional chemotherapy are limited, and the prognosis remains poor. Based on some promising preclinical results, HDACis grew the expectations of being a considerable alternative treatment for these tumors with the limited options in the metastatic context. The preliminary clinical studies confirmed that HDACis could inhibit tumor growth, activate specific molecular pathways and act synergistically with other already approved treatments, such as 131I-MIBG. However, more data are required to prove the benefit of their use, to determine the specific HDACis compound that should be used in the rare indication of metastatic PCCs/PGLs, and to detect the optimal dosing and the way of administration (as monotherapy or in combination with other agents), following in parallel their safety profile.

## Data Availability

Data supporting this article are included within the reference list. Please contact corresponding author for any further information.
